# Phytoplasma-Responsive microRNAs Modulate Hormonal, Nutritional, and Stress Signalling Pathways in Mexican Lime Trees

**DOI:** 10.1371/journal.pone.0066372

**Published:** 2013-06-18

**Authors:** Farveh Ehya, Aboozar Monavarfeshani, Ehsan Mohseni Fard, Laleh Karimi Farsad, Mojtaba Khayam Nekouei, Mohsen Mardi, Ghasem Hosseini Salekdeh

**Affiliations:** 1 Department of Genomics, Agricultural Biotechnology Research Institute of Iran, Karaj, Tehran, Iran; 2 Department of Systems Biology, Agricultural Biotechnology Research Institute of Iran, Karaj, Tehran, Iran; 3 Department of Molecular Systems Biology, Cell Science Research Center, Royan Institute for Stem Cell Biology and Technology, ACECR, Tehran, Iran; BASF Cropdesign, Belgium

## Abstract

**Background:**

Witches’ broom disease of Mexican lime (*Citrus aurantifolia* L.), which is associated to the phytoplasma ‘*Candidatus Phytoplasma aurantifolia*’, is a devastating disease that results in significant economic losses. Plants adapt to biotic stresses by regulating gene expression at the transcriptional and post-transcriptional levels. MicroRNAs (miRNAs) are a recently identified family of molecules that regulate plant responses to environmental stresses through post-transcriptional gene silencing.

**Methods:**

Using a high-throughput approach to sequence small RNAs, we compared the expression profiles of miRNAs in healthy Mexican lime trees and in plants infected with ‘*Ca. P. aurantifolia*’.

**Results:**

Our results demonstrated the involvement of different miRNAs in the response of Mexican lime trees to infection by ‘*Ca. P. aurantifolia*’. We identified miRNA families that are expressed differentially upon infection with phytoplasmas. Most of the miRNAs had variants with small sequence variations (isomiRs), which are expressed differentially in response to pathogen infection.

**Conclusions:**

It is likely that the miRNAs that are expressed differentially in healthy and phytoplasma-infected Mexican lime trees are involved in coordinating the regulation of hormonal, nutritional, and stress signalling pathways, and the complex interactions between them. Future research to elucidate the roles of these miRNAs should improve our understanding of the level of diversity of specific plant responses to phytoplasmas.

## Introduction

Witches’ broom disease of Mexican lime (*Citrus aurantifolia* L.) is a devastating disease that results in significant economic losses [1]. It is associated to ‘*Candidatus Phytoplasma aurantifolia*’, an obligate biotrophic plant pathogen. Since it was first reported in the northern coastal plain of the Sultanate of Oman in the 1980s, witches’ broom disease has spread throughout the region and has also affected Mexican lime trees in southern Iran [2], [3]. Between 1997 and 2008, the number of trees infected with witches’ broom disease increased from 51,000 to 500,000, and the disease made it necessary to destroy many hectares of cultivated trees in Iran [4]. The symptoms of witches’ broom disease include the development of many thin secondary shoots, each with shortened internodes, which develop from axillary buds that normally stay dormant. Many of these shoots, the so-called ‘witches’ brooms’, appear during the advanced stages of the disease. The leaves of infected trees become dry, and the trees eventually collapse within 4 or 5 years after infection.

Although significant progress has been made in understanding the signalling processes that are involved in several plant–pathogen interactions [5], knowledge about the molecular mechanisms involved in the pathogenicity of phytoplasmas and the symptoms that they evoke in host plants has been limited primarily by an inability to culture phytoplasmas *in vitro* [6].

Transcriptomic and proteomic analyses of Mexican lime trees infected with ‘*Ca. P. aurantifolia*’ have identified several candidate genes and proteins that might be involved in the interaction of Mexican lime trees with the phytoplasma, and thus have provided new insights into the interaction of this pathogen with its host [7], [8]. Recently, microRNAs (miRNAs) have emerged as a new family of regulatory molecules that are involved in the control of plant development and responses to abiotic and biotic stresses through post-transcriptional gene silencing [9]. MicroRNAs are 21–24-nucleotide (nt) noncoding RNAs that are derived from precursors called premiRNAs, each of which is approximately 50–350 nt [10]. It has been suggested that miRNAs are involved in pathogen-associated molecular patterning (PAMP)-triggered immunity (PTI) and the disease resistance response that is induced by plant pathogens [9], [11]. Upon pathogen infection, host plants can recognize the nature of the pathogen on the basis of pathogen-associated molecular patterns, and induce a defence response that involves rapid changes in levels of hormones, metabolites, and gene expression.

The first miRNA that was determined to play a role in plant PTI (miR393) was reported by Navarro et al. in 2006, and regulates plant PTI by negatively regulating auxin signalling [12]. Expression of miR393, which is induced by the bacterial PAMP peptide flg22, represses the expression of auxin receptors, including the transport inhibitor response 1 (TIR1) protein [12]. The notion that miRNAs function in plant pathogen defences was supported further by the demonstration that miRNA-deficient *Arabidopsis thaliana* (Arabidopsis) mutants support enhanced growth of the bacterium *Pseudomonas syringae* pv tomato (Pst) DC3000 *hrcC*, as well as several bacterial strains that are not pathogenic to Arabidopsis, including *Pseudomonas syringae pv. phaseolicola*, *Pseudomonas fluorescens*, and *Escherichia coli* [13].

Many conserved plant miRNAs have been detected using conventional cloning and Sanger sequencing [14], although only highly expressed miRNAs can be identified readily with these techniques. Computational analysis of available ESTs has also been applied to predict a limited number of miRNAs [15], [16]. Recently, next-generation sequencing technologies, including 454 and Illumina sequencing, have become popular tools for miRNA discovery owing to their abilities to identify and quantify a large number of miRNAs [15].

In the study reported herein, we used high-throughput sequencing to identify miRNAs that are expressed differentially in Mexican lime trees in response to infection by ‘*Ca. P. aurantifolia*’. The results of the study improve our understanding of the molecular basis of the progression of witches’ broom disease, and might enable us to identify new strategies to control the disease.

## Materials and Methods

### Plant Material

We prepared one-year-old Mexican lime trees by growing healthy seedlings in a greenhouse at 25°C–28°C. Then obtained healthy trees were grafted using cuttings from either healthy or ‘*Ca. P. aurantifolia*’-infected Mexican lime trees. All of the grafted plants were arranged randomly in the greenhouse and covered with plastic bags for 1 month to increase the humidity in their immediate environment. Twenty weeks after inoculation, the branches of six healthy and six infected plants were harvested, frozen immediately in liquid nitrogen, and stored at –80°C until further use. Phytoplasma infection was detected by nested PCR as described previously [8]. The 16S rRNA region of the phytoplasma DNA was amplified by PCR using the P1 [17] and P7 [18] primers. The resulting P1–P7 amplicons were then used as template DNA for nested-PCR amplification with the universal primer pair for phytoplasma 16S rRNA, R16F2n/R2 [19]. The purified PCR products were sequenced and the phytoplasma strains were classified using iPhyClassifier, as described by Zhao et al. [20].

### RNA Extraction and Sequencing

The RNA from 100-mg samples of leaves from six infected trees and six healthy trees was isolated using the High Pure miRNA Isolation Kit (Roche, Germany) in accordance with the manufacturer's instructions, and quantified using an ND-1000 spectrophotometer (NanoDrop Thermo Scientific, USA). The pooled miRNAs from the infected and from the healthy tissues were used to construct libraries of small RNAs, and sequencing was performed using an Illumina HiSeq2000 instrument (Beijing Genomics Institute, China), following standard protocols.

### Data Analysis

The 50-nt sequence tags that were obtained from the HiSeq sequencing were subjected to a data-cleaning procedure that included the removal of low-quality tags, adaptors, and reads shorter than 18-nt. The remaining sequence tags were then annotated as belonging to different categories (rRNA, snRNA, siRNA, snoRNA, or tRNA) by performing a BLASTn (version 2.2.24+) search against the Rfam (http://www.sanger.ac.uk/software/Rfam) and NCBI (http://www.ncbi.nlm.nih.gov/) databases.

To identify conserved miRNAs, the remaining unique small RNA reads were aligned with the known plant miRNAs in the miRBase database [21] using BLASTn (version 2.2.24+). Only sequences with fewer than three mismatches were regarded as known miRNAs. To identify potential novel miRNAs and their precursor sequences, the remaining unannotated reads were aligned with non-coding unigenes of in-house *C. aurantifolia* Transcriptome database (deposited in SRA database with Accession no. SRA058604) using Mireap (http://sourceforge.net/projects/mireap/).

The normalized miRNA expression level (The actual count of miRNA/Total count of clean reads*1000000) was used to compare the differential expression of miRNAs in healthy and infected trees. All small RNAs with a read count less than 10 were excluded from further analysis. We considered a fold-change ≥1.5 and p≤0.05 as our criteria to identify differentially expressed miRNAs. P-value were calculated based on Poisson distribution [22].

Targets of the miRNAs were also predicted using psRNATarget [23] by submitting the miRNA sequences to a search against our in-house trascriptome database of *C. aurantifolia*.

### Stem-loop Real-time PCR Analysis to Validate miRNA Expression Patterns

All cDNAs were synthesized using an RNA template and reverse transcriptase (Invitrogen, USA). Primers for stem-loop RT-PCR and gene-specific real-time PCR primers (Table S1) for five miRNAs were designed according to Chen et al. [24]. Real-time PCR was performed with an iCycler iQ Real-Time PCR System (Bio-Rad, USA) and an iQ SYBR Green Supermix Kit (Bio-Rad), 50 ng of cDNA, and 10 pM of each forward and reverse gene-specific primer in a total volume of 25 µl. Cycling conditions consisted of a denaturation step at 94°C for 2 minutes, followed by 35 cycles at 94°C for 30 seconds, 50°C for 1 minute to anneal the optimized primers, and 72°C for 90 seconds. Melting curve analysis was performed after each RT-PCR run to verify the specificity of the SYBR Green dye, and the absence of primer-dimers. The Mexican lime 18S rRNA was used as an internal control. Transcript levels were determined from the data collected during real-time PCR experiments by using a P-value of ΔΔCT ≤0.05 and a confidence interval of α = 0.01.

### Indole-3-acetic Acid Measurement

High-performance liquid chromatography (HPLC) was used to purify plant hormones from leaf tissues of six healthy and six infected plants. Extraction and partial purification were performed as described previously [25]. The liquid chromatography system from Shimadzu Corporation (Kyoto, Japan), equipped with a 250 mm × 4.6 mm, 5-µm Hypersil ODS (C_18_) HPLC column from Altech, was used. The mobile phase consisted of a 15-min isocratic elution with a 50∶50 (v/v) mixture of 0.2% acetic acid and 100% methanol delivered at a flow rate of 0.70 ml/min. The mobile phase was degassed immediately before use. The column temperature was 40°C and the absorbance of the eluate was monitored at 257 nm. The retention times for indole-3-acetic acid (IAA) in this system was approximately 8. 3 minutes. The levels of compounds separated by HPLC were quantified on the basis of their peak areas. The results were analyzed using t-test. The level of significance was set at P≤0.05.

## Results

### High-throughput Sequencing of *Citrus aurantifolia* Small RNAs

To investigate the roles of small RNAs from the Mexican lime tree in response to witches’ broom disease, two small RNA libraries, from healthy and infected trees, were constructed and sequenced using Illumina HiSeq2000 high-throughput sequencing. The numbers of raw reads for infected and healthy samples were 17,996,992 and 14,069,882, respectively. After the output reads had been cleaned and nucleotides 18–30 nt long selected, we identified 2,751,072 unique sequences from healthy plants and 3,304,749 unique sequences from infected plants. The raw small RNA data were submitted to Gene Expression Omnibus (GEO) under accession no. GSE44279.

### Identification of Known miRNAs

**Figure 1:**
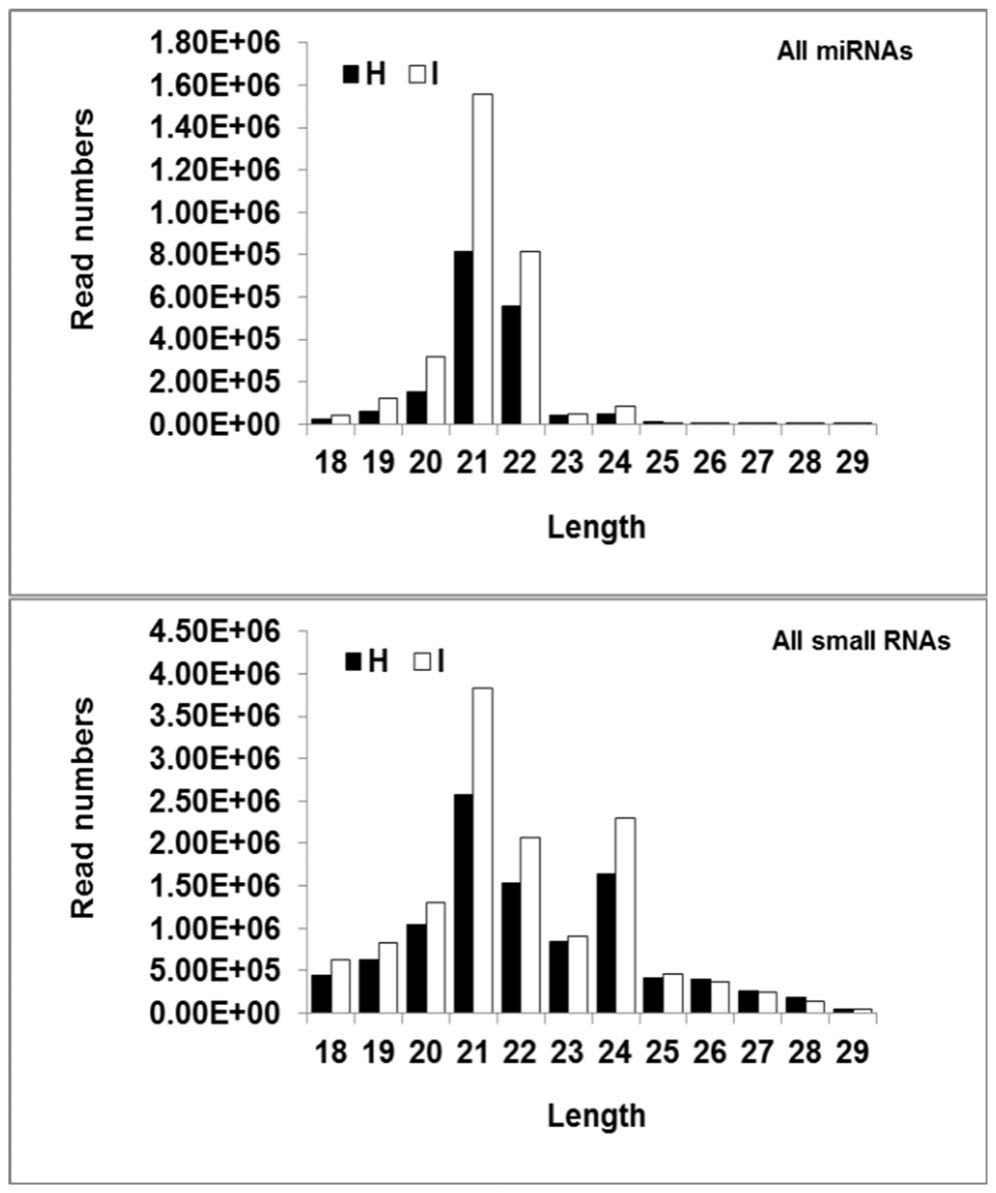
The size distribution of both total small RNA and miRNA sequences that were identified in Healthy (H) and Phytoplasma-infected (I) plants. Whereas most of the small RNAs were either 21 nt or 24 nt, most of the miRNAs were either 21 nt or 22 nt.

To identify known miRNAs, all the sequences obtained after the data-cleaning procedure from both healthy and infected plants were used to interrogate the in-house *C. aurantifolia* EST sequences and citrus ESTs in the NCBI databases to identify orthologous and paralogous miRNA sequences with an identity of at least 18 nt, and no more than 3 mismatched nt. The matching sequences were then subjected to a search against Rfam and protein database to exclude rRNAs, tRNAs, and degradation products from noncoding and coding sequences. The remaining small RNA tag sequences were aligned with the miRNA precursors/mature miRNAs of the corresponding species in miRBase16.0. Percentage reads mapping to different categories was shown in Figure S1. We investigated the size distribution of both the total small RNA and miRNA sequences that were identified. Whereas most small RNA sequences were either 21 or 24 nt long, most miRNAs were either 21 or 22 nt long (Figure 1).

### Sequence Variations in miRNAs

The sequences of many of the miRNAs that were identified from Mexican lime varied from their reference sequences in miRBase, which generated multiple mature variants that we hereafter refer to as isomiRs. We also observed isomiRs for star miRNA sequences in both of the libraries. Star miRNAs are small RNAs processed from the hairpin arms that are complementary to those that encode mature miRNAs. It has been shown that isomiRs are not artifacts generated during massively parallel sequencing [26], [27].

Among the 12,148 known miRNA variants (Table S2) that were identified in the present study, 1,474 variants (Table S3) corresponded to 151 known miRNAs (Table S4) that were represented by at least 10 transcripts in at least one of our two small RNA libraries. For those miRNAs that had isomiRs, the number of different isomiRs ranged from one (18 miRNA) to 213 (the miR3948), with three miRNA families each having more than one hundred types of isomiR. Seventy two miRNAs had no isomiRs.

In some cases, the most abundant sequence did not correspond exactly to the miRNA sequence deposited in miRBase 16.0 (Table S2). For example, we found that a variant of miR156c with one mismatch relative to the reference sequence deposited in miRBase 16.0 was more abundant than the variant that matched the reference sequence exactly (30,869 and 49,016 reads vs. 144 and 224 reads in infected and healthy samples, respectively).

The most abundant variant of miR166k in the two libraries (116 and 141 reads in infected and healthy samples, respectively) was 18 nt long, whereas the number of reads for the 20-nt miR166k sequence that corresponded to the reference sequence was only 12 and 11 in infected and healthy samples, respectively. Differences in miRNA processing under different physiological conditions might account for the differences in the abundances of isomiRs.

### Identification of Novel Citrus miRNAs

Novel miRNAs were predicted using Mireap (https://sourceforge.net/projects/mireap/), which analyses the secondary structure, Dicer cleavage site, and minimum free energy of unannotated small RNA tags. We identified 47 candidate novel miRNAs from the Mexican lime tree (Table S5). Among these, 28 miRNAs were represented by at least 10 transcripts in one of our two libraries, with the largest number of reads (49,014) reported for miRIH11 (Table 1). The lengths of the newly identified miRNAs ranged from 20–23 nt, and the negative folding free energies varied from –67.7 to –18.8 kcal mol^−1^. We also identified 35 star miRNAs in the libraries. The novel miRNAs were less abundant than the conserved miRNAs.

**Table 1:** Novel miRNA identified in this study: H: healthy plant; I: infected plant.

	ID	Raw Count I	Raw Count H	FC	P value	ARM	Sequence	MFE
**Common novel miRNA candidate**	miR-IH1	9	17	0.672	0.00627	5p	TGTCTGCGGCTAAATTTAACC	−27.2
	miR-IH3	44	43	0.791	0.021811	5p	TGCAGGTGAGATGATACCGTCA	−38.4
	miR-IH4	255	273	0.736	0.000023	3p	TGGTTGATGCCTGAGAGTCGA	−39.73
	miR-IH5	129	119	0.83	0.009551	5p	AAGGGATAAGTTGGAGGGAGC	−36.1
	miR-IH6	29	10	1.078	0.003918	3p	AGGTGCAGTTGCAAGTGCAGA	−65.92
	miR-IH7	21479	10450	1.603	0	5p	CGAAGGTCCGAGGTCGAGGTT	−47.96
	miR-IH8	733	952	0.609	0	3p	ACGGGTATGGAAAAGGGGCGCAT	−52.2
	miR-IH9	5797	1747	2.553	0	5p	AAGCGACGTGCAGTTGGGGCT	−32.2
	miR-IH10	111	67	1.103	0.006508	3p	TGCTCGCTCCTCTTCTGTCAGC	−49.9
	miR-IH11	49041	37466	1.023	0.000004	5p	GGTCATGGGAGGATTGGCGAGA	−56.3
**I specific novel miRNA candidate**	miR-I1	29	0	1.348	0	5p	CCTTGGGTGAGCTGGTGGGGGC	−43.22
	miR-I3	14	0	1.055	0.000135	5p	TTTTGTTGCATGATGCTGATAA	−35.6
	miR-I6	10	0	0.977	0.00136	3p	CACTTGACTTGTGAAGTATGGTA	−55.3
	miR-I7	266	0	5.98	0	3p	GAAAGTGAATGTTGGCGGTTG	−20.2
	miR-I10	13	0	1.035	0.00024	3p	TGGGAGGCTGCACCTTACATT	−36.4
	miR-I11	121	0	3.146	0	5p	TGGCTGAGAAGATAGGATGCC	−28.8
	miR-I13	268	0	6.019	0	3p	TTGAGCCGCGTCAATATCTCC	−41.24
	miR-I14	31	0	1.387	0	3p	TGAGGTTCTTGGGGAGAGTAG	−44.86
	miR-I16	12	0	1.016	0.000429	3p	TTAGTGATGTGATGGAGCCGG	−27.1
	miR-I18	10	0	0.977	0.00136	5p	TTCTGACTTGAAGGAAGTGCT	−23.7
	miR-I19	2986	0	59.14	0	5p	AATGGGTGCGTGGGCAAGAGA	−37.73
**H specific novel miRNA candidate**	miR-H1	0	53	0.336	0	5p	TTGCTGGTGATGTGGTGTTG	−35.84
	miR-H2	0	23	0.496	0	3p	CGGTTACTGCCTGAGAGTCGA	−34.5
	miR-H5	0	175	0.145	0	3p	TTTCTCTTATCGTTATCTGTG	−53.57
	miR-H6	0	12	0.601	0.000022	5p	CTTGCTTTCTGACTTGCTTAA	−67.7
	miR-H7	0	39	0.395	0	5p	AGGTATTGGCGCGCCTCAATT	−38.4
	miR-H9	0	11	0.613	0.000051	3p	GAGGGAGAGAGAGAGAGAGAGAG	−30.4
	miR-H11	0	10	0.625	0.000116	5p	TCAGACTTGAGATTTTCAACGAC	−18.8

### Response of Mexican Lime miRNAs to Witches’ Broom Disease of Lime

**Figure 2:**
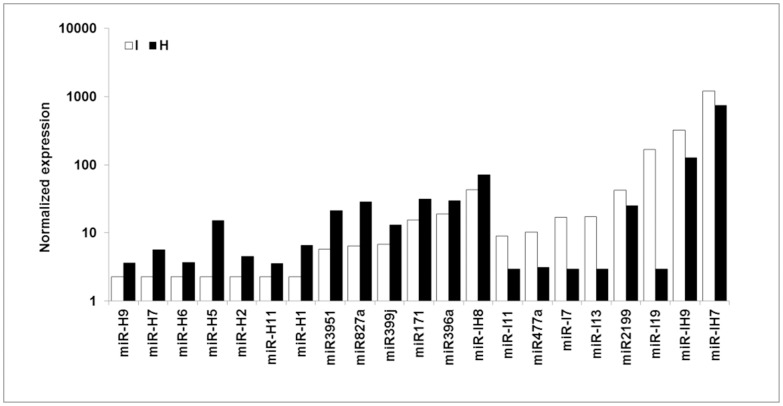
The normalized expression value of group I of miRNAs in healthy (H) and phytoplasma-infected (I) plants.

Comparison of the abundances of miRNAs in infected and healthy plants enabled us to identify three major groups of miRNAs that were expressed differentially in response to infection with ‘*Ca. P. aurantifolia’*. In the first of these groups (I), There is just one variant for each miRNA. This group is composed of 7 known and 14 novel miRNAs. Of these, the abundances of 2 known and 6 novel miRNAs increased after phytoplasma infection, whereas the abundances of 5 known and 8 novel miRNAs decreased after phytoplasma infection (Figure 2 and Table S6).

**Figure 3:**
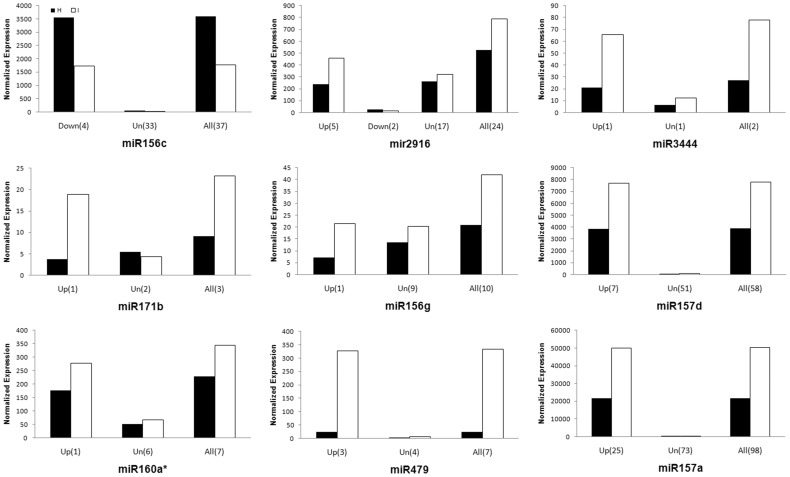
The normalized expression value of some members of group II in healthy (H) and phytoplasma-infected (I) plants. Up, Down and Un indicate upregulated, downregulated and unchenaged expression pattern, respectively. All indicates the overall expression of all variants for a miRNA. Numbers in bracket are the number of variants.

In group II, the overall abundances of all isomiRs for each miRNA also either increased or decreased significantly in response to phytoplasma infection. However, some isomiRs of each miRNA in this group either showed no significant change or even the opposite expression pattern (miR2916) to the other isomiRs of the same miRNA. In most cases, the overall trend of regulation for the group of isomiRs is consistent with the pattern of regulation of the most abundant isomiR (Figure 3 and Table S7). In two cases, miRNA 390b and miR473, different variants showed no significant expression change but the overall trend was upregulation. The group II miRNAs comprised 23 known miRNAs, of which 14 were more abundant and 9 were less abundant in infected plants when compared with healthy plants.

The overall abundances of members of the third group of miRNAs (group III) did not change significantly in response to phytoplasma infection, although some variants were either more or less abundant in infected plants compare to healthy plants (Table S4 and S8). This group comprised 121 known and 14 novel miRNAs, with 2 miRNAs that contained upregulated isomiRs, and 9 miRNA that contained downregulated isomiRs. In addition, 5 miRNA contained both up- and downregulated isomiRs beside unchanged variants.

1177 unigenes derived from our transcriptome database were predicted as potential targets for 113 miRNAs which has one or more differentially expressed isomiRs (Tables S9 and S10). Previously, upregulation and downregulation of some potential targets for downregulated and upregulated miRNAs, respectively, has been reported in RNA [8] and protein [7] level.

The respective abundances of serine-type peptidase and glutaminyl-tRNA synthetase proteins, which are potential targets for miR157a and miR390b (both upregulated following infection of Mexican lime tree with *‘Ca. P. aurantifolia’*) were approximately three- and two-fold lower in infected plants than in healthy plants. Also, pectin methylesterase and monocopper oxidase-like protein which are potential targets for downregulated miR2911 and miR159a, respectively, upregulated at the protein level [7]. Moreover, upregulated miR157a might be involved in the downregulation of its target, a serine/threonine protein kinase which showed to be downregulated in the phytoplasma-infected plant at the mRNA level [8].

Generally, identified targets for differentially expressed miRNAs mainly influenced metabolism, stress and defense responses, proteolysis, oxidation-reduction process and signalling pathways which are consistent with biological processes previously reported to be affected by infection of Mexican lime tree with *‘Ca. P. aurantifolia’* [7], [8].

We found that the expression of several star miRNAs, such as miR160d*, miR157d*, miR156f*, miR169c*, and miR157a*, also changed in response to phytoplasma infection. These results raise questions about the functions and targets of star miRNAs. Close examination of the potential miRNA–target RNA pairs indicated that several star miRNAs were associated with potential targets (Tables S9 and S10). Therefore, it is possible that some star miRNAs might lead to the cleavage of mRNAs, in a manner identical or similar to that regulated by mature miRNAs [28].

**Figure 4:**
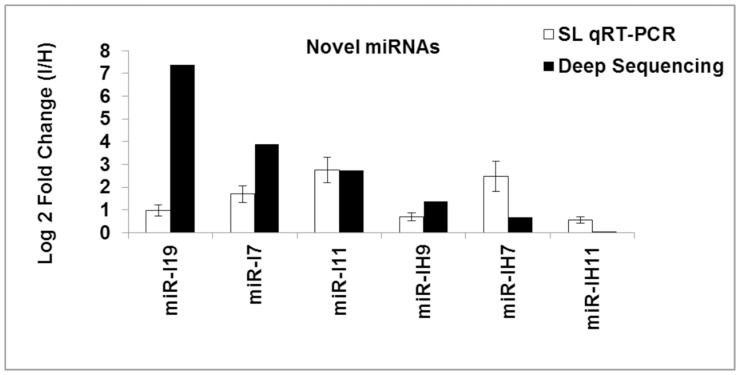
Effects of ‘*Ca. P. aurantifolia’* infection on miRNA relative expression evaluated by qRT-PCR. To validate the results of the deep sequencing, we used qPCR to analyse the expression of 5 novel miRNAs. SL: Stem Loop.

To validate the results of the deep sequencing, we used qPCR to analyse the expression of 5 novel miRNAs. Both methods generated similar results for all miRNAs. Slight differences between the results from Illumina deep sequencing and qRT–PCR can possibly be attributed to differences between the two profiling techniques (Figure 4).

## Discussion

The response of plants to biotic stress is a complex process that involves many genes and a range of biochemical and molecular mechanisms. Plant adaptation to stress is achieved through the regulation of gene expression at the transcriptional and post-transcriptional levels. Recently, miRNAs have emerged as a family of regulatory molecules that control plant development and responses to environmental stress through post-transcriptional gene silencing. We used high-throughput sequencing of small RNAs to compare the expression profiles of miRNAs in healthy and phytoplasma-infected Mexican lime trees.

### miRNA-mediated Auxin Signalling Might Regulate the Response of the Mexican Lime Tree to Phytoplasma

The important plant hormone auxin is involved in many plant processes, including cell elongation and growth. Repression of auxin receptor genes by overexpression of miR393, which targets auxin receptors, increases the resistance of Arabidopsis to the bacteria Pst DC3000, whereas activation of auxin signalling enhances the susceptibility of Arabidopsis to Pst DC3000 [12]. These results suggest that auxin promotes susceptibility to bacterial disease. We also observed that the level of IAA significantly increased (up to 5 fold) in phytoplasma-infected Mexican lime trees compared with healthy trees (Figure S2). The substantial increase in IAA levels that we observed in response to phytoplasma infection might reflect a susceptibility reaction of Mexican lime trees to phytoplasma. We also observed changes in the expression of several isomiRs of miRNAs involved in auxin signalling, such as miR160 (for miR160*), miR166 and miR167 (Table S3).

Previous studies revealed that the effects of miR160, miR166, miR167, and miR393 on auxin signalling might lie at the level of regulation of auxin response factors (ARFs), which belong to a family of transcription factors that play an important role in the early auxin response [29]. In addition, auxin responsive elements (AuxREs) are often present in the promoters of miRNA genes, such as miR167. Therefore, feedback regulation might be prevalent in miRNA-mediated auxin signalling pathways in plants, and a feedback control mechanism might exist in other regulatory or signal transduction pathways controlled by miRNAs [30]. Levels of miR159, which targets for degradation the mRNAs that encode MYB transcription factors, are lower in phytoplasma-infected plants than in healthy plants [31]. Members of the MYB superfamily play a role in plant defence against pathogens and plant hormone responses [32]. It has been suggested that MYBs redirect auxin signal transduction by interacting with the Carboxyl termini of ARFs [33].

Therefore, taken together with the findings obtained using different plant species, our findings suggest that auxin pathways that are affected by miRNAs could play important roles in the responses of Mexican lime trees to phytoplasma infection.

### Cross-talk between Auxin Signalling and Nutrient Signalling

**Figure 5:**
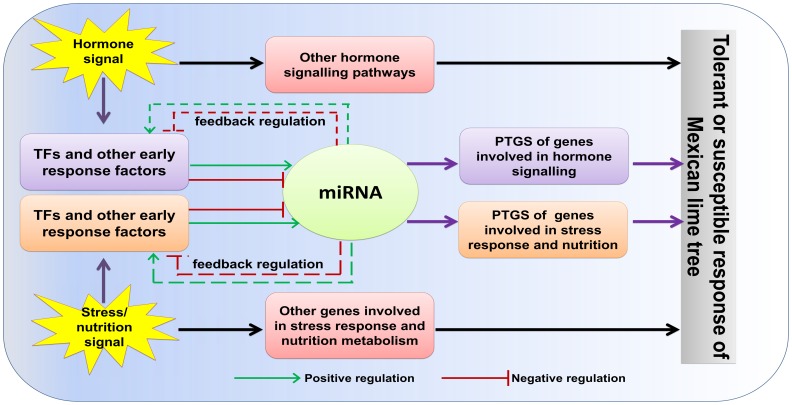
A model of the miRNA-mediated interaction of hormone, nutrient, and stress signalling that is involved during the interaction of the Mexican lime tree with phytoplasma. Our results suggest that biotic and abiotic stress signals converge upstream of the events that control the abundances of miRNAs that were expressed differentially in response to infection with ‘*Ca. P. aurantifolia*.

A reduction in metabolic rate is a survival strategy that is used commonly by plants to divert energy and other resources to adaptive mechanisms that help plants to cope with stress conditions. Auxin signalling is crucial for plant growth and development, as well as plant responses to nutritional deficiency and other forms of stress. The observation that the levels of miRNAs that target TIR1 and several ARFs are affected by nutrient deficiency and other stresses suggests a role for miRNAs in the regulation of auxin responses in response to environmental stress [9]. For instance, the levels of expression of miR160, miR167, and miR393 change in response to ABA application, exposure to salt, drought, cold, and heat stresses, and to changes in sulphate metabolism in a range of plant species. The same three miRNAs are also induced in response to bacterial infection [33]. These observations suggest that biotic and abiotic stress signals converge upstream of the events that control the abundances of these miRNAs. A model of the miRNA-mediated interaction of hormone, nutrient, and stress signalling that is involved during the interaction of the Mexican lime tree with phytoplasma is shown in Figure 5. However, further experiments are required to test the validity of the model and uncover the molecular events that underlie the response of the Mexican lime tree to phytoplasmas.

### Phytoplasma Infection Might Induce miRNA-mediated Regulation of Brassinosteroid Biosynthesis

The expression level of different miR156 family members altered in our study in response to phytoplasma infection. The abundance of miR156 was found to decrease after treatment with brassinosteroids, but the other phytohormones tested had no effect [34]. Furthermore, several genes that might be involved in brassinosteroid biosynthesis or signalling were affected by overexpression of miR156 [34]. Brassinosteroids, a class of polyhydroxysteroid, are involved in a broad spectrum of cellular and physiological processes, including stem elongation, root inhibition, fruit development, pollen tube growth, xylem differentiation, photosynthesis, ethylene biosynthesis, proton pump activity, and gene expression [35], [36]. In addition, brassinosteroids can induce plant tolerance to a variety of biotic and abiotic stresses. It has been demonstrated that hydrogen peroxide mediates the transcriptional induction of defence or antioxidant genes by brassinosteroids. Following perception of the brassinosteroid signal, NADPH oxidase might be activated to produce reactive oxygen species (ROS), which initiate a protein phosphorylation cascade [37].

### Levels of Several miRNAs Responsive to Biotic and Abiotic Stresses Change after Phytoplasma Infection

Given their need to cope with continuous exposure to various abiotic and biotic stresses in their natural environment, plants have evolved sophisticated mechanisms to sense and respond to environmental conditions. The generation of ROS is a key process that is common to both biotic and abiotic stresses [38]. Production of ROS in plants in response to phytoplasma infection has also been reported [39]–[41]. Phytohormones constitute another class of endogenous molecules that modulate the protective responses of plants to biotic and abiotic stresses, primarily via signalling crosstalk [42]. Biotic and abiotic stresses modulate the expression of both different and overlapping sets of genes [43]. Several miRNAs that were found to be expressed differentially in healthy and phytoplasma-infected lime trees in the present study were reported previously to be present at different levels in other species after exposure to both abiotic and biotic stresses. Combined with these studies, our findings thus further support the notion of crosstalk between various environmental stresses at the level of molecular signal transduction.

It has been suggested that stress-regulated miRNAs might be regulated by stress-related transcription factors. In Arabidopsis, the promoters of 11 miRNAs, namely, miR156, miR169, miR171, miR393, miR399, miR439, miR535, miR806, miR809, miR812, and miR815, contained all of the stress-related cis-elements investigated, including the drought-responsive element, low temperature-induced element, MYC-binding site, and MYB-binding site [28]. The predicted targets of miR394 are F-box proteins with different functions. Exposure to either drought or salt stress decreases the abundance of miR530-3p. This suggests that the cold-responsive promoter elements upstream of certain miRNA genes repress miRNA expression during drought and salt stresses to discriminate between different abiotic stresses [44]. The ability of drought and salt stress to increase miR530-3p abundance, and the ability of cold stress to decrease miR530-3p abundance highlights the need for more in-depth and detailed characterization of stress-responsive miRNAs in plants.

Exposure of Arabidopsis leaves to the virulent form of the bacterium Pst DC3000 increases the abundances of miR393, miR319, miR158, miR160, miR167, miR166, and miR159, while decreasing the abundances of miR390, miR408, and miR398 [33]. The expression levels of many stress-related genes are affected by changes in miRNAs. For example, overexpression of miR156 changes the expression of several drought- or disease-responsive genes, such as dehydration-responsive element-binding proteins, NAC transcription factors, and pathogenesis-related protein 1 [34]. Exposure of Arabidopsis to drought decreases the abundance of miR169 and increases the abundance of its target mRNA, which encodes the transcription factor NFYA5. Furthermore, whereas miR169-overexpressing plants are drought sensitive, NFYA5-overexpressing transgenic lines are drought tolerant [45].

### The Role of microRNAs in Nutrient Homeostasis

Plants need at least 14 essential mineral elements to keep ensure the normal growth and development and required to ensure the completion of their life cycles. Several miRNAs have emerged as being essential for the fine regulation and optimization of nutrient homeostasis and plant adaptation to low nutrient concentrations [9]. The genomes of the phytoplasmas are extremely reduced, and lack many of the genes that encode essential metabolic pathways in other organisms. This highly specialized nutritional strategy, which typifies biotrophic plant pathogens such as phytoplasmas, probably involves the diversion of host-cell metabolism to support pathogen survival and compatibility [46].

Several miRNAs that were identified as phytoplasma-responsive in the present study are known to play a role in nutrient homeostasis. For instance, miR395 regulates the accumulation and allocation of sulphate in Arabidopsis by targeting members of the ATP sulphurylase and sulphate transporter 2 protein families, both of which control sulphate metabolism [47]. Besides miR395, levels of miR156, miR160, miR164, miR167, miR168, and miR394 are also altered during sulphate deprivation in *Brassica napa*, which suggests their possible contribution to modulation of the necessary growth and developmental adjustments that are required for adaptation to sulphate-deprived conditions [48].

In addition, the abundance of miR156, miR169, miR395, and miR399* is also altered during phosphate deprivation, which suggests a role for other miRNAs in phosphate homeostasis [49], [50]. Of these miRNAs, the role of miR399 during phosphate deprivation has been well characterized. It has been shown that upregulation of miR399 negatively regulates the expression of an E2 ubiquitin-conjugating enzyme, which in turn negatively regulates mobilization of internal phosphate from older to younger leaves [51]. Both miR397 and miR857 were also shown to be upregulated under conditions of copper deficiency and are predicted to target transcripts that encode the Cu-containing laccases and plantacyanin [10]. It has been suggested that under Cu deficiency, miR397 and miR857 might decrease the expression of nonessential Cu-containing proteins, and consequently increase the availability of copper for essential plastocyanin-related functions.

### The Architecture of Mexican Lime Trees Might be Modified by Upregulation of miRNA157

The shoots of phytoplasma-infected Mexican lime trees have compact structures with very small, pale green leaves. These so-called witches’ brooms have many thin secondary shoots, which have shortened internodes and develop from axillary buds that normally stay dormant.

Phytoplasma effectors contribute to the symptoms characteristic of witches’ broom disease in plants infected with ‘*Ca. P. aurantifolia’*. A 4.5-kDa effector protein encoded by the *TENGU* gene of onion yellows phytoplasma induced witches’ broom and dwarfism in *Nicotiana benthamiana*. Transgenic Arabidopsis lines that express *TENGU* have symptoms of witches’ broom disease [52]. It has been suggested that SAP11, which is one of the 56 effectors of aster yellows phytoplasma, might induce symptoms of witches’ broom diseases [53]. Transgenic Arabidopsis plants that expressed *SAP11* showed an increased number of axillary stems and curly leaves–a phenotype that resembles the symptoms of witches’ broom disease in plants infected with aster yellows phytoplasma.

Phytoplasma effectors can alter plant morphology by manipulating signalling pathways in plants [52]. We observed that the expression of several variants of miR157 increased in response to phytoplasma infection. Overexpression of miR157 from Arabidopsis in torenia (*Torenia fournieri*) induces a high degree of branching and the formation of small leaves–a phenotype that resembles that associated with the effects of miR157 overexpression in other species [54]. The apparent target-sequence specificity of miR157 appears to enable fine regulation of the expression of the gene that encodes the SQUAMOSA-promoter binding protein (SBP). Our results indicate that miR157 could play a role in modifying the architecture and leaf morphology of the Mexican lime tree in response to phytoplasma infection.

### Conclusions

The results that we have presented here demonstrate the involvement of different miRNAs in the response of the Mexican lime tree to infection with ‘*Ca. P. aurantifolia’*. Further investigation of the role of these miRNAs promises to enhance our understanding of the diversity and specificity of particular plant responses to phytoplasma. These responses involve changes in the regulation of signalling pathways that are activated in the leaves of Mexican lime trees by plant hormones, changes in nutrient status, and exposure to different biotic and abiotic stresses. An important future challenge would be to understand further how these responses are coordinated. Furthermore, a complete understanding of the actions of miRNAs depends on the identification of the target genes. The identification of entire sets of miRNAs and their targets will lay the foundation that is needed to unravel the complex miRNA-mediated regulatory networks and will greatly increase our understanding of plant tolerance to biotic and abiotic stresses. This may facilitate efforts to design plants that are able to resist stress without compromising their yields.

## Supporting Information

Figure S1 **Percentage of different categories of small RNA libraries; Phytoplasma-infected (I), and Healthy (H) Mexican lime trees.**
(TIF)

Figure S2 **High performance liquid chromatography (HPLC) for identification and quantification of auxin and abscisic acid plant hormones.**
(TIF)

Table S1 **The list of stem-loop RT-PCR primers for five miRNAs.**
(XLSX)

Table S2 **The list of 12148 miRNA variants detected in our study.**
(XLSX)

Table S3 **The list of 1474 variants of known miRNAs which had 10 transcripts or more in at least one library.**
(XLSX)

Table S4 **The list 151 conserved miRNA families which had 10 transcripts or more in at least one library.**
(XLSX)

Table S5 **Novel miRNA identified in this study.**
(XLSX)

Table S6 **The list of miRNAs in group I and their variants expression patterns.**
(XLSX)

Table S7 **The list of miRNAs in group II and their variants expression patterns.**
(XLSX)

Table S8 **The list of miRNAs in group III and their variants expression patterns.**
(XLSX)

Table S9 **Up- and downregulated miRNAs of group I and II and their targets.**
(XLSX)

Table S10 **Complete list of all groups (groups I, II and III) miRNAs and their targets.**
(XLSX)
