# Supplementary figures and images for: Phytoplasma-Responsive microRNAs Modulate Hormonal, Nutritional, and Stress Signalling Pathways in Mexican Lime Trees
Source: PLoS One. 2013 Jun 18;8(6):e66372. doi: 10.1371/journal.pone.0066372 (PMC3688891; doi:10.1371/journal.pone.0066372)

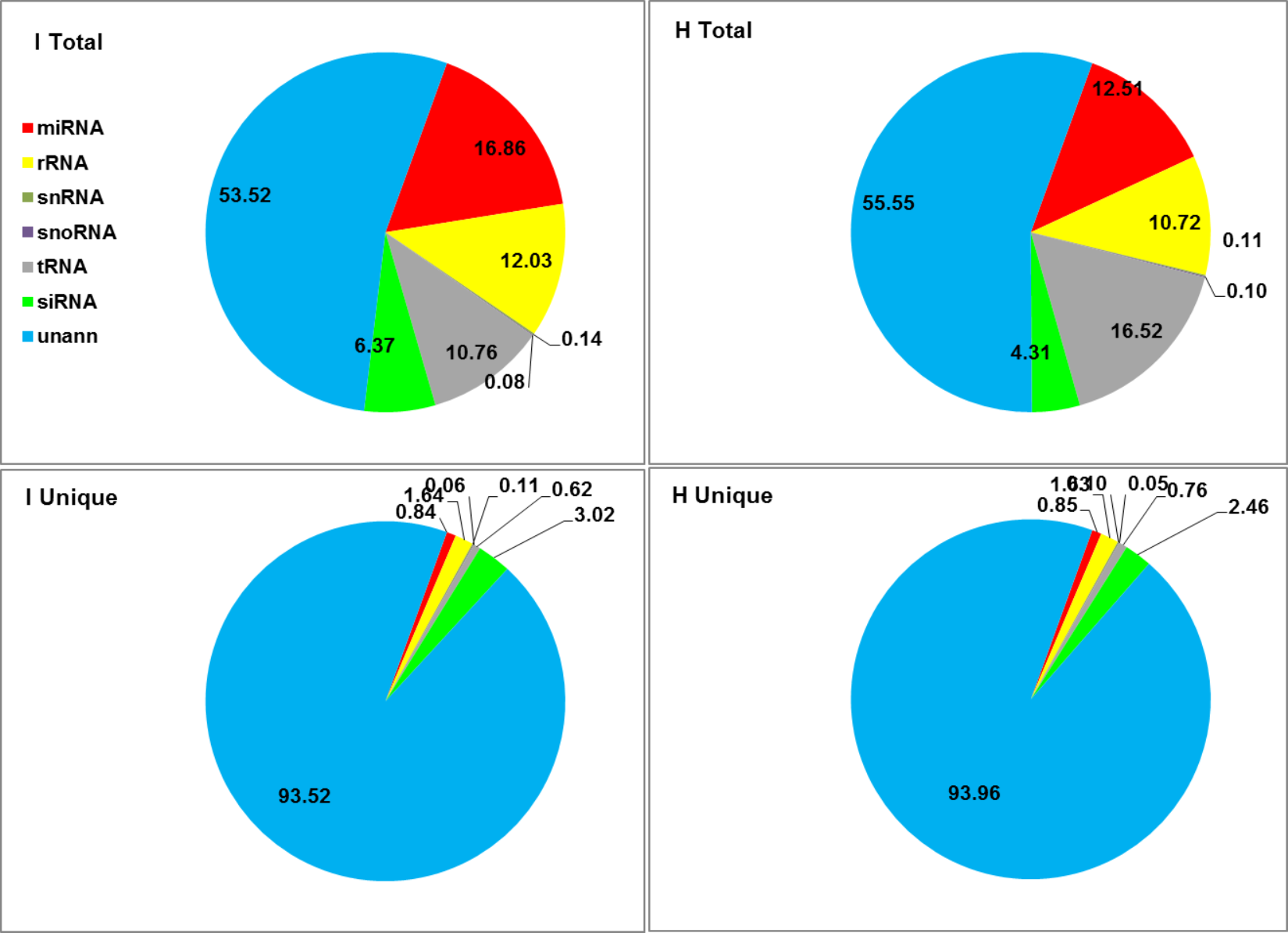

Supplement: Figure S1 — Percentage of different categories of small RNA libraries; Phytoplasma-infected (I), and Healthy (H) Mexican lime trees. (TIF) [file pone.0066372.s001.tif]
